# Synthesis of terpinyl acetate from α-pinene catalyzed by α-hydroxycarboxylic acid–boric acid composite catalyst

**DOI:** 10.1371/journal.pone.0299218

**Published:** 2024-04-25

**Authors:** Zhonglei Meng, Rongxiu Qin, Rusi Wen, Junkang Xie, Guiqing Li, Yonghong Zhou

**Affiliations:** 1 Guangxi Key Laboratory of Superior Timber Trees Resource Cultivation/Guangxi Forestry Research Institute, Nanning, China; 2 Institute of Chemical Industry of Forest Products, CAF Nanjing, Nanjing, China; Vietnam Academy of Science and Technology, VIET NAM

## Abstract

To enhance the yield of the one-step synthesis of terpinyl acetate from α-pinene and acetic acid, this study evaluated α-hydroxycarboxylic acid (HCA)–boric acid composite catalysts based on orthogonal experimental design. The most important factor affecting the terpinyl acetate content in the product was the HCA content. The catalytic performance of the composite catalyst was related to the pKa1 of HCA. The tartaric acid–boric acid composite catalyst showed the highest catalytic activity. The α-pinene conversion reached 91.8%, and the terpinyl acetate selectivity reached 45.6%. When boric acid was replaced with B2O3, the HCA composite catalyst activity was enhanced, which reduced the use of HCA. When the lactic acid and B2O3 content accounted for 10% and 4% of the α-pinene mass content, respectively, the α-pinene conversion reached 93.2%, and the terpinyl acetate selectivity reached up to 47.1%. In addition, the presence of water was unfavorable to HCA–boric acid composite catalyst. However, a water content less than 1% of the α-pinene mass content improved the catalytic activity of HCA–B2O3. When the tartaric acid–B2O3 was used as catalyst, and the water content was 1% of the α-pinene mass content, the α-pinene conversion was 89.6%, and the terpinyl acetate selectivity was 47.5%.

## Introduction

Terpinyl acetate exists naturally in a variety of essential oils, e.g., cedarwood oil, pine needle oil, melaleuca oil, and cardamom oil. It is a colorless to slightly yellowish liquid with lemon and lavender aromas. It is one of the twenty most important flavor industry chemicals.

In manufacturing, terpinyl acetate can be synthesized through the reaction between α-pinene and acetic acid, but the terpinyl acetate selectivity is low. Gainsford et al. performed the reaction at room temperature for 24 h using H-beta zeolite as the catalyst, and the terpinyl acetate yield was 29% [1]. Yamanaka used Fe2(SO4)3 as a catalyst to react limonene/dipentene with acetic acid to produce terpinyl acetate, but they did not report the yield of the product [2]. Liu et al. carried out the reaction using an ionic liquid as the catalyst, and the highest yield of terpinyl acetate was 35.7% [3, 4]. Machado et al. used sulfa SBA-15 as a catalyst, and the yield of terpinyl acetate was 23% [5]. Golets et al. used an ion exchange resin as the catalyst, and the terpinyl acetate yield was 35% [6]. Wijayati et al. used natural zeolite as the catalyst and obtained a terpinyl acetate yield of 21.4% [7]. However, when pinene, acetic anhydride, and water were used as the raw materials, di-chloromethane as the solvent, and Y-type zeolite as the catalyst, Wijayati et al. obtained a terpinyl acetate yield of 52.8% [8]. The disadvantage of this reaction is that the raw material acetic anhydride cannot be recycled, and the added dichloromethane may cause environmental pollution.

Therefore, we aimed to improve the low yield for terpinyl acetate by evaluating different catalysts and optimizing the reaction conditions in order to enhance the pinene conversion and terpinyl acetate selectivity. In our previous study [9] on pinene hydration, citric acid and phosphoric acid (85%) were used as composite catalysts to study the reaction of pinene with acetic acid. The content of terpinyl acetate in the product was 14.1% [9]. Considering that the water contained in phosphoric acid may not be conducive to the formation of terpinyl acetate, we also used anhydrous citric acid and P2O5 as composite catalysts, but a large number of polymers were generated during the reaction.

Boric acid has low toxicity, and as an additive, it promoted the conversion of wood into biochar and significantly increased carbon yield [10]. A catalyst composed of boric acid and acetic anhydride catalyzed the reaction of pinene with acetic acid, and the esterification products were fenvalerate acetate, bornyl acetate, and terpinyl acetate. Their mass ratio was about 1:2:3, and the total yield of the three esters was about 70% [11]. Because of the electrical absorption of the boron atom, boric acid can form complexes with compounds containing hydroxyls, and it forms 1:1 and 1:2 complexes with glycolic and tartaric acids [12]. When tartaric acid and boric acid form complexes, the conductivity and optical rotation of the complexes increase [13, 14]. The structure, reaction equilibrium, reaction kinetics, and thermodynamics of the complex formed by boric acid and α-hydroxycarboxylic acid (HCA) have been studied [15–17]. These studies showed that the dehydration reaction of boric acid and HCA will form a stable five-membered ring complex [18].

Boric acid can increase the concentration of hydrogen ions when added to aqueous HCA solution [19]; therefore, it may form a composite catalyst with HCA. This study screened HCA–boric composite catalysts through orthogonal experiments with the goal of identifying a catalyst that can enhance the terpinyl acetate yield.

## Results and discussion

### Screening of HCA–boric acid composite catalyst

To identify a catalyst that can improve the yield of terpinyl acetate, lactic acid, glycolic acid, mandelic acid, tartaric acid, and citric acid were used individually to form composite catalysts with boric acid. Five variables, namely acetic acid content, HCA content, boric acid content, temperature, and time, were evaluated at four different levels, as shown in Table 1. The α-pinene conversion, terpinyl acetate content, and terpinyl acetate selectivity were set as dependent variables. A set of orthogonal experiments with five factors at four levels (L16(45)) were conducted to understand the catalytic performance of different catalysts.

**Table 1:** Orthogonal experiment levels.

Factor	Levels			
	1	2	3	4
A: Temperature (°C)	22	27	33	40
B: Time (h)	15	20	24	30
C: Molar ratio of acetic acid to α-pinene [n(acetate acid):n(α-pinene)]	1:1	2:1	3:1	4:1
D: Mass ratio of HCA to α-pinene [m(HCA):m(α-pinene)]	2:50	3:50	1:10	1:5
E: Mass ratio of boric acid to α-pinene [m(boric acid):m(α-pinene)]	1:500	1:250	7:1000	1:100

#### Orthogonal experiment results

Five-factor, four-level orthogonal experiments were designed to investigate the effects of various factors on alpha-pinene conversion and terpinyl acetate selectivity. The reaction between α-pinene and acetic acid is usually accompanied by isomerization and ring-expansion side reactions. By-products, such as camphene, limonene, and terpinene, are generated from isomerization, and α-pinene acetate and bornyl acetate are generated by ring-expansion. Therefore, the α-pinene conversion and terpinyl acetate selectivity were used as indicators to evaluate the reaction, as shown in the supporting information (S1–S5 Tables in S1 File). When the α-pinene purity is constant, the content of terpinyl acetate determined by gas chromatography (GC) can be used directly as an index. The α-pinene used in this study was from the same batch; therefore, the GC-determined content of terpinyl acetate was used as the catalyst-screening indicator. The range value (R) of each factor was calculated and are presented in Table 2. K1–K4 in the table are the test indexes at the four experimental levels for the factors temperature, time, acetic acid dosage, HCA dosage, and boric acid dosage. In addition, k1–k4 are the average values of K1–K4, respectively.

**Table 2:** Results of orthogonal experiments on the tartaric acid–boric acid catalyst.

No.	A: Temperature (°C)	B: Time (h)	C: n(acetate acid):n(α-pinene)	D: m(tartaric acid):(α-pinene)	E: m(boric acid):(α-pinene)	Terpinyl acetate content (%)
1	27	20	2:1	3:50	1:500	10.5
2	27	30	3:1	1:50	7:1000	0.7
3	22	20	3:1	1:5	1:250	41.0
4	33	24	3:1	1:10	1:500	31.8
5	22	15	1:1	1:50	1:500	0.2
6	22	30	2:1	1:10	1:100	31.7
7	33	15	2:1	1:5	7:1000	22.4
8	22	24	4:1	3:50	7:1000	32.5
9	40	24	2:1	1:50	1:250	12.4
10	27	24	1:1	1:5	1:100	2.6
11	33	30	1:1	3:50	1:250	13.7
12	27	15	4:1	1:10	1:250	37.7
13	40	15	3:1	3:50	1:100	28.5
14	33	20	4:1	1:50	1:100	14.8
15	40	30	4:1	1:5	1:500	10.7
16	40	20	1:1	1:10	7:1000	19.3
K1	51.449	85.630	77.026	85.200	53.172	
K2	105.504	56.785	101.917	28.061	74.857	
K3	82.658	79.277	35.755	76.715	104.835	
K4	70.846	88.765	95.759	120.481	77.593	
k1	12.862	21.408	19.257	21.300	13.293	
k2	26.376	14.196	25.479	7.015	18.714	
k3	20.665	19.819	8.939	19.179	26.209	
k4	17.712	22.191	23.940	30.120	19.398	
Range (R)	13.514	7.995	16.541	23.105	12.916	
Rank	3	5	2	1	4	

Note: K represents the sum of the test results of the corresponding factor at a specified level. For example, in the column for factor A, K1 represents the sum of the results at the first level of the factor. k represents the average of the test results for the corresponding factor at a specified level. For example, in the column for factor A, k1 represents the average of the results for the first level of the A factor.

Since the evaluation index was the content of terpinyl acetate, a higher K value indicates better catalytic performance. According to the K values of each factor, the best scheme could be identified. In Table 2, the preferred scheme of tartaric acid–boronic acid was: as follows reaction temperature of 22°C, reaction time 30 h, a molar ratio of acetic acid to α-pinene of 2, a mass ratio of tartaric acid to α-pinene of 0.2, and a mass ratio of boric acid to α-pinene of 0.7% (A2B4C2D4E3). The influence of each factor was ranked as D > C > A > E > B.

From S2 Table in S1 File, the preferred scheme of the lactic acid–boronic acid composite catalyst was as follows: reaction temperature of 40°C, reaction time of 20 h, molar ratio of acetic acid to α-pinene of 4, mass ratio of lactic acid to α-pinene of 0.1, and mass ratio of boric acid to α-pinene of 0.4% (A4B2C4D3E2). The influence of each factor was ranked as D > A > B > E > C.

In S3 Table in S1 File, the preferred scheme of glycolic acid–boronic acid composite catalyst was as follows: reaction temperature of 40°C, reaction time of 30 h, molar ratio of acetic acid to α-pinene of 4, mass ratio of glycolic acid to α-pinene of 0.1, and mass ratio of boric acid to α-pinene of 0.2% (A4B4C4D3E1). The influence of each factor was ranked as D > A > C > B > E.

From S4 Table in S1 File, the preferred scheme of mandelic–boric acid composite catalyst was as follows: reaction temperature of 33°C, reaction time of 30 h, molar ratio of acetic acid to α-pinene of 2, mass ratio of mandelic acid to α-pinene of 0.1, and mass ratio of boric acid to α-pinene of 0.7% (A3B4C2D3E3). The influence of each factor was ranked as D > B > C > A > E.

In S5 Table in S1 File, the preferred scheme of citric acid–boronic acid composite catalyst was as follows: reaction temperature of 40°C, reaction time 20 h, molar ratio of acetic acid to α-pinene of 4, mass ratio of citric acid to α-pinene of 0.1, and mass ratio of boric acid to α-pinene of 0.2% (A4B2C4D3E1). The influence of each factor was ranked as D > A > B > E > C.

The R value, calculated from the orthogonal experimental results, indicated the most important factor affecting the terpinyl acetate content was the amount of HCA. For the three catalysts associated with lactic acid, glycolic acid, and citric acid, the second most important factor was the reaction temperature. For the catalyst associated with mandelic acid, the second most important factor was the reaction time. For the catalyst associated with tartaric acid, the second most important factor was the amount of acetic acid.

The orthogonal experimental results also indicated boric acid content had minimal impact on the terpinyl acetate content. Boric acid could combine with the hydroxyl group in HCA to form a ligand, thereby facilitating HCA to donate protons. It became necessary to further investigate the impact of the amount of boric acid and HCA using single-factor experiments.

#### Catalytic performance analysis of the composite catalysts

The scheme selected according to the size of the K values was verified. The contents of terpinyl acetate in the products corresponding to the catalysts lactic acid–boric acid, glycolic acid–boric acid, mandelic acid–boric acid, tartaric acid–boric acid and citric acid–boric acid were 12%, 17%, 17%, 24%, and 13%, respectively. Because the content of terpinyl acetate in the scheme was lower than the optimal result in the orthogonal experiment, the scheme corresponding to the optimal orthogonal experiment result was the better scheme.

The optimal results obtained from the orthogonal experiments with different catalysts are summarized in Table 3, where the tartaric acid–boric acid composite catalyst showed the best catalytic effect. The highest terpinyl acetate content in the product was 41.0%. The catalytic performance of the composite catalyst from high to low followed the trend: tartaric acid–boric acid > glycolic acid–boric acid > mandelic acid–boric acid > citric acid–boric acid > lactic acid–boric acid.

**Table 3:** Optimal results in orthogonal experiments of different composite catalysts.

No.	A: Temperat-ure (°C)	B: Time (h)	C: n(acetic acid):n(α-pinene)	D: m(HCA):m(α-pinene)	E: m(boric acid):m(α-pinene)	Terpinyl acetate content (%)
1	22	15	3:1	Tartaric acid 1:5	1:250	41.0
2	40	30	4:1	Lactic acid 1:5	7:1000	25.1
3	40	15	4:1	Glycolic acid 1:5	1:100	31.3
4	33	15	3:1	Mandelic acid 1:5	1:250	30.3
5	40	30	4:1	Citric acid 1:5	1:500	27.8

As shown in Table 3, the optimal results of different composite catalysts corresponded with different reaction temperature, reaction time, boric acid content, and acetic acid content. However, when the HCA to α-pinene mass ratio was 1:5, the product had the highest level of terpinyl acetate content across all experiments. The corresponding acetic acid content was the highest (4:1) and second highest (3:1), indicating excess acetic acid was favorable to the generation of terpinyl acetate.

The catalytic performance of the composite catalyst was related to the acidity coefficient of HCA, i.e., the smaller the acidity coefficient, the better the catalytic performance. The acidity coefficient (pKa1) of HCA is presented in supporting information (S6 Table in S1 File). As shown in Table 3, the catalytic performance of the composite catalyst was negatively correlated with the pKa1 of HCA, except for citric acid. The acidity coefficient of tartaric acid was the smallest, and the terpinyl acetate content of the associated 16 experiments ranged from 2.6% to 41.0%. The content in five of the experiments was above 30%. Therefore, the performance of tartaric acid-associated catalysts was deemed optimal. The terpinyl acetate content of composite catalysts associated with citric acid, glycolic acid, mandelic acid, and lactic acid were distributed within 1.8% to 27.8%, 2.2% to 31.3%, 1.7% to 30.3%, 1.4% to 25.1%, respectively. Their corresponding maximum content was all below that of tartaric acid.

**Fig 1:**
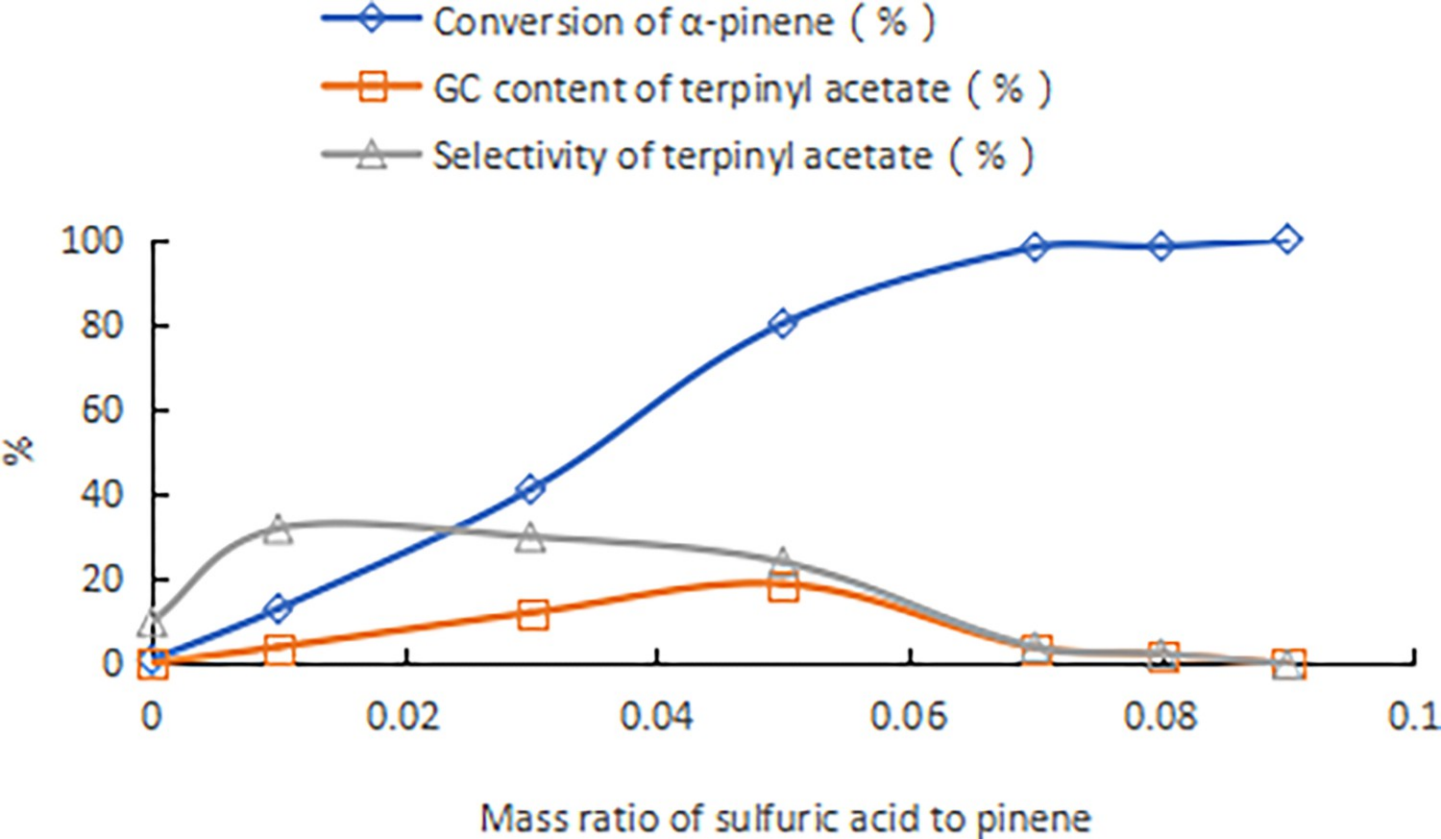
Ethylation of α-pinene catalyzed by sulfuric acid (98% concentration).

Using sulfuric acid with a concentration of 98% as a comparison, the mass ratio of α-pinene to acetic acid was 1:2.5, the reaction temperature was 25°C, and the reaction time was 24 h. The results are shown in Fig 1. With an increase of the sulfuric acid dose, the conversion rate of α-pinene gradually increased, and the content and selectivity of terpinyl acetate in the product first increased and then decreased. When the amount of sulfuric acid was 9% of the mass of α-pinene, no terpinyl acetate was detected in the product. The acetates in the product were fenvalerate acetate, isoborneol acetate, and borneol acetate, with contents of 1.9%, 8.3%, and 8.8%, respectively. In addition, the product was about 25% polymer. The highest terpinyl acetate content as determined by GC in the product was 18.9%. From comparative experiments, it was seen that although the composite catalyst composed of boric acid and HCA improved the acidity of the catalyst, the increase in acidity was not the only factor that improved the catalytic activity.

### Optimization of the composite catalysts

#### Effect of compositional changes on catalytic performance

When the reaction temperature was in the range of 22°C to 40°C and the reaction time ranged from 15 h to 30 h, the catalytic performance of a single catalyst was poor, with the α-pinene conversion below 10%. Terpinol acetate cannot be prepared without a catalyst; without one, the yield of terpinol acetate is close to 0.

**Fig 2:**
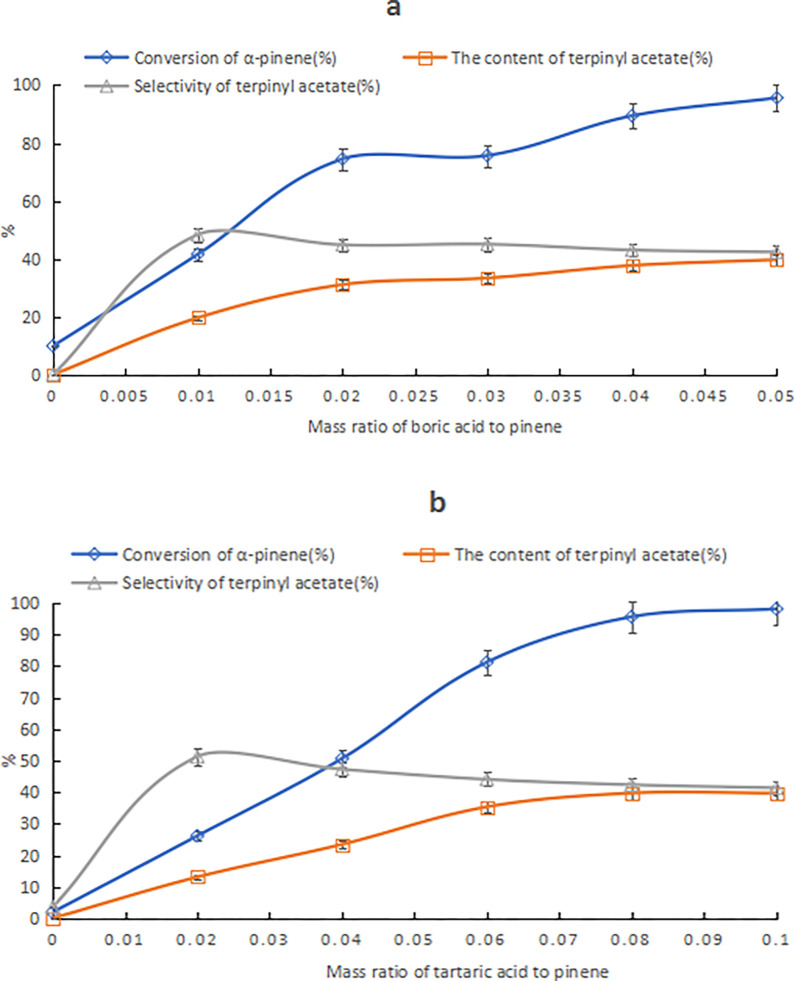
The effect of compositional changes of tartaric acid–boric acid catalyst on products. Note: a) Effect of boric acid changes; conditions: α-pinene, acetic acid, tartaric acid mass ratio of 10:25:0.8 and reaction temperature of 25°C for 24 h. b) Effect of tartaric acid changes; conditions: α-pinene, acetic acid, tartaric acid mass ratio of 10:25:0.5 and reaction temperature of 25°C for 24 h.

As shown in Fig 2, regardless of whether tartaric acid or boric acid was used, the catalytic performance of a single catalyst was poor; however, boric acid can react with a hydroxyl group in HCA, such as tartaric acid, to form complexes with high catalytic ability. In Fig 2A, as the amount of boric acid increased, the α-pinene conversion and terpinyl acetate content increased, while the terpinyl acetate selectivity first increased and then decreased gradually. In Fig 2B, as the tartaric content increased, the α-pinene conversion increased rapidly, while the terpinyl acetate content increased first and then leveled off. Tartaric acid and boric acid mutually enhanced their catalytic performances. Therefore, the ratio of the two can be adjusted to screen and optimize catalysts. When the boric acid amount increased to 5% of the α-pinene mass content and the tartaric acid was 8% of the α-pinene mass content, the terpinyl acetate content in the product was as high as 39.8%, as presented in Fig 2B. This result was close to the optimal result from the orthogonal experiments in Table 3, but it required much less tartaric acid. The total catalyst usage was reduced from 20.4% to 13%.

#### Replacing boric acid with boric anhydride

**Fig 3:**
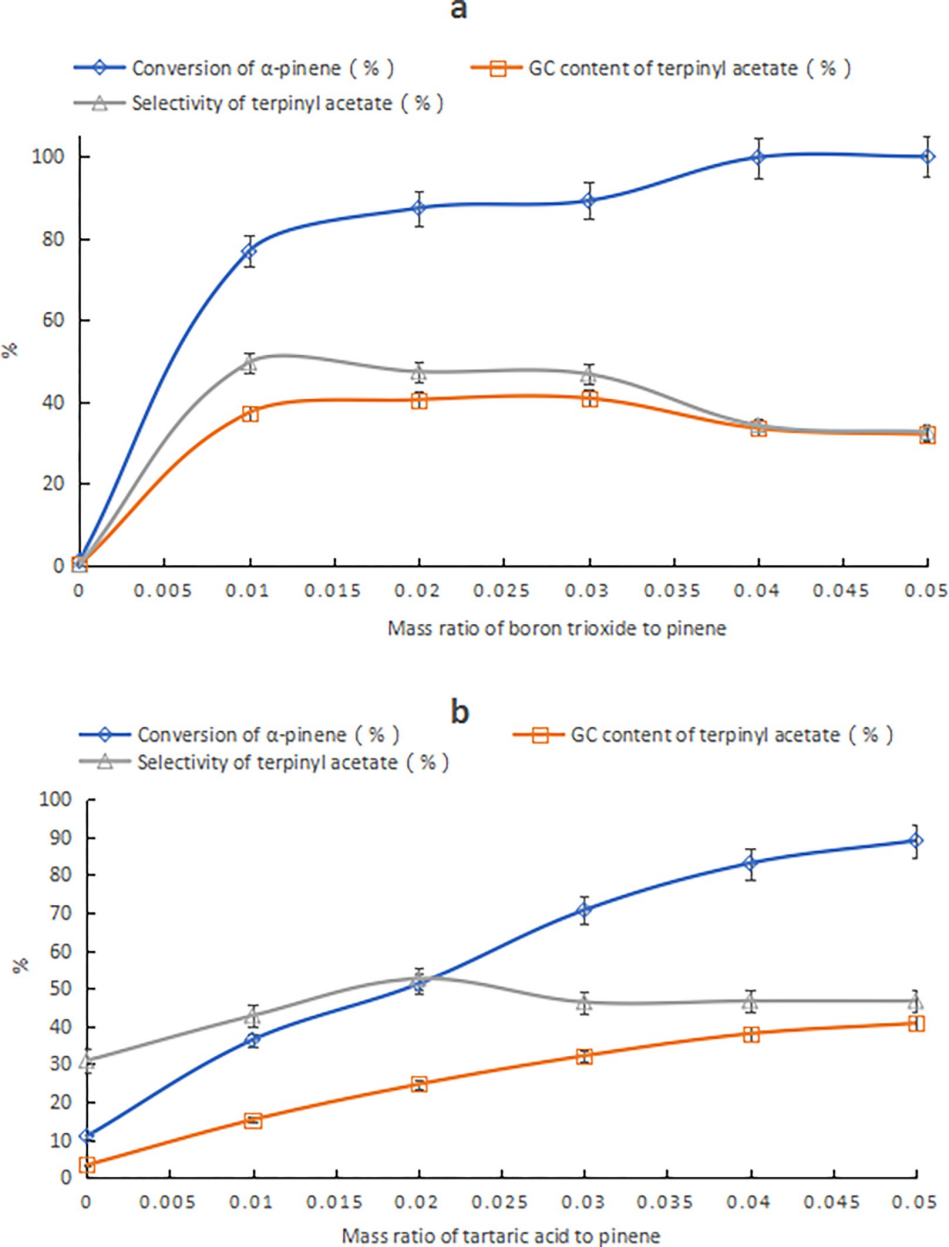
The effect of compositional changes of tartaric acid–B2O3 catalyst on products. Note: a) Effect of B2O3 changes; conditions: α-pinene, acetic acid, tartaric acid mass ratio of 10:25:0.5 and reaction temperature of 25°C for 24 h. b) Effect of tartaric acid changes; conditions: α-pinene, acetic acid, B2O3 mass ratio of 10:25:0.3 and reaction temperature of 25°C for 24 h.

Boric acid will form a coordination bond with the hydroxyl group of HCA through the dehydration reaction, and boric anhydride (B2O3) is more likely to react than boric acid. Thus, the use of B2O3, instead of boric acid, improved the composite catalyst reactivity, as shown in Figs 5 and 6. In Fig 3A, as the B2O3 amount increased, the α-pinene conversion increased, while the terpinyl acetate content and selectivity increased first then decreased. The decrease in terpinyl acetate selectivity was due to the increase of bornyl acetate by-product. In Fig 3B, the α-pinene conversion and the terpinyl acetate content both increased gradually as the tartaric acid amount increased, while the terpinyl acetate selectivity first increased and then decreased. When the tartaric acid content was above 5%, the isomerization reaction was accelerated, and by-products, such as limonene, terpinene, and α-terpinene, were formed. When the tartaric acid content was above or equal to 8%, a large amount of polymer was produced, and bornyl acetate was formed instead of terpinyl acetate. The suitable composition of the tartaric acid–B2O3 catalyst that favored the production of terpinyl acetate was B2O3 content at 1%–3% of the α-pinene mass and tartaric acid content at 4%–5% of the α-pinene mass. When the tartaric acid and B2O3 content was 5% and 3% of the α-pinene mass, respectively, the content of terpinyl acetate was 40.8%. This result was close to the optimal results shown in Table 3, with the added advantage that the amount of composite catalyst could be reduced from 20.4% to 8% of the α-pinene mass.

**Fig 4:**
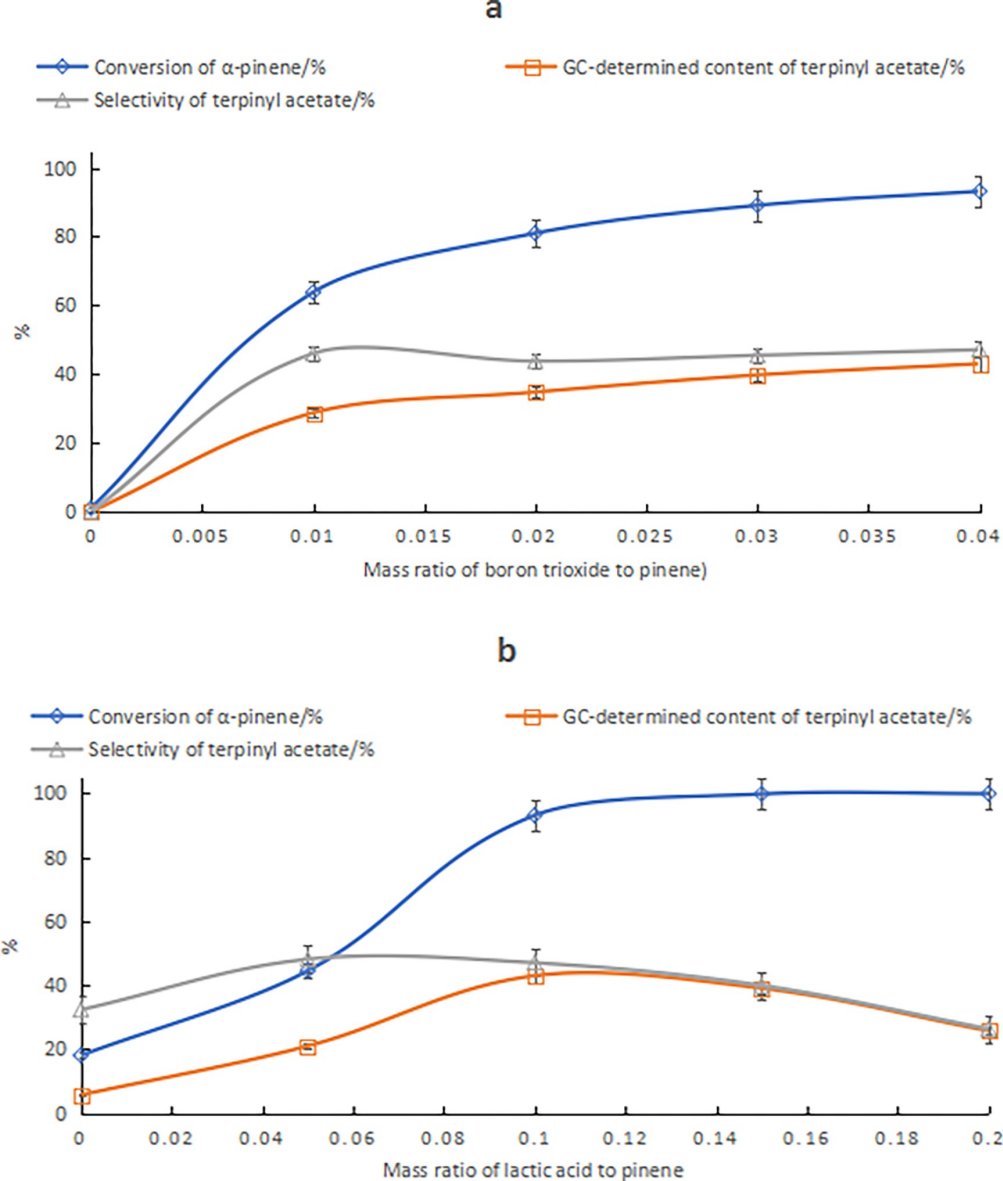
The effect of compositional changes of lactic acid–B2O3 catalyst on product. Note: a) Effect of B2O3 changes; conditions: α-pinene, acetic acid, lactic acid mass ratio of 10:25:1 and reaction temperature of 25°C for 24 h. b) Effect of lactic acid changes; conditions: α-pinene, acetic acid, B2O3 mass ratio of 10:25:0.4, and a reaction temperature of 25°C for 24 h.

Although the lactic acid–boric acid catalyst showed the least favorable catalytic performance, its activity was greatly enhanced by replacing boric acid with B2O3. For lactic acid–B2O3 composite catalysts, the α-pinene conversion increased gradually as the B2O3 amount increased, while the terpinyl acetate content increased gradually, while the selectivity decreased, as shown in Fig 4A. As the lactic acid content increased, the α-pinene conversion increased gradually, whereas the terpinyl acetate content and selectivity increased first and then decreased, as shown in Fig 4B. When the lactic acid and B2O3 content was 10% and 4% of the α-pinene mass, respectively, the terpinyl acetate content was 43.0%. Compared to the optimal results in Table 3, the terpinyl acetate content increased by 17.9%, while the amount of composite catalyst decreased from 20.7% to 14% of the α-pinene mass. In contrast, when B2O3 was used in place of boric acid, the impact on citric acid-associated catalysts was negligible.

**Fig 5:**
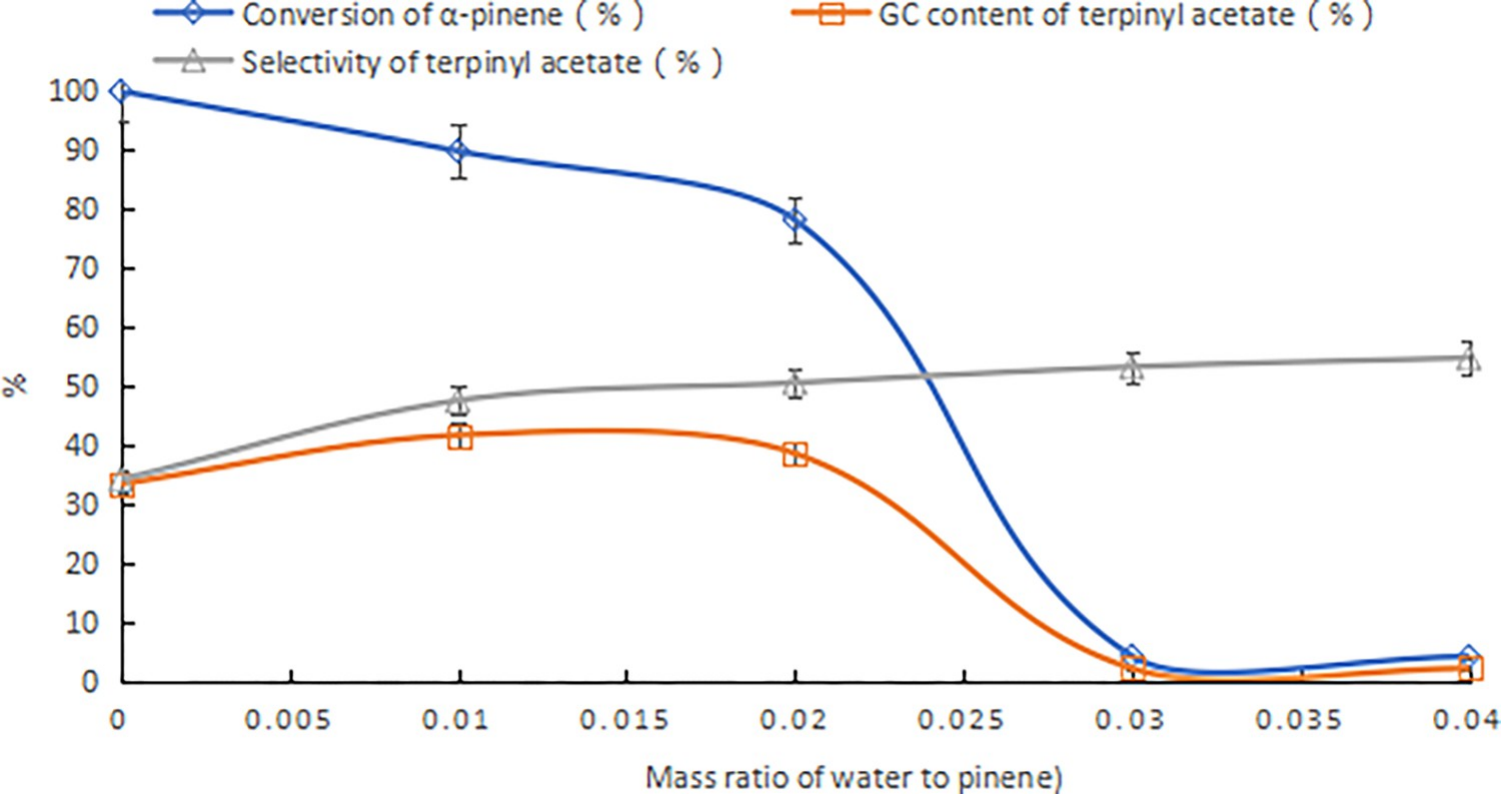
The effect of water on tartaric acid–B2O3 composite catalyst. Conditions: α-pinene, acetic acid, tartaric acid and B2O3 mass ratio of 10:25:0.5:0.4 and reaction temperature of 25°C for 24 h.

In the synthesis of terpinyl acetate from α-pinene, presence of water has a negative impact on the HCA–boric acid composite catalyst. However, a small amount of water can help improve the selectivity of HCA–B2O3, as shown in Fig 5. As the water content increased, the α-pinene conversion decreased. Moreover, when the water content was above 2% of the α-pinene mass content, the α-pinene conversion decreased rapidly, while the terpinyl acetate selectivity increased first then leveled off. The terpinyl acetate content increased first and then decreased rapidly. This was because the excess water negatively affected the miscibility between acetic acid and α-pinene, which hindered the reaction mass transfer and reduced the reaction rate of α-pinene. When the α-pinene conversion and terpinyl acetate selectivity are considered collectively, an acceptable reaction performance was reached when the water was 1% of the α-pinene mass content. The α-pinene conversion was 89.6%, and terpinyl acetate content and selectivity were 41.7% and 47.5%, respectively.

### Changes in α-pinene and terpinyl acetate content with reaction time

**Fig 6:**
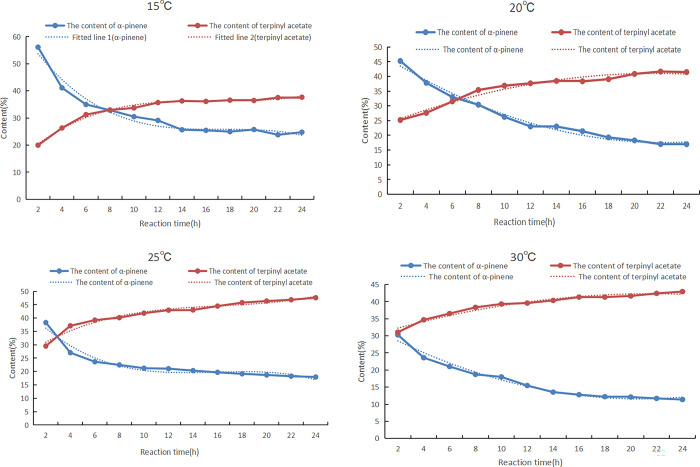
The changes in the contents of α-pinene and terpinyl acetate in the product with increasing reaction time. The mass ratio between α-pinene, acetic acid, tartaric acid, and boric anhydride was 10:25:0.5:0.4.

During the esterification reaction of α-pinene with acetic acid, the products generated were camphene, ramine, terpinene, isoterpinene, and other isomeric by-products, as well as fenvalerate acetate, bornyl acetate, and other ester by-products. The reaction rate and the time to reach equilibrium could be determined by measuring the change in the content of α-pinene and terpinyl acetate in the product with the reaction time. Using tartaric acid and boric acid as composite catalysts, the changes in α-pinene and terpinyl acetate in the product with increasing reaction time were investigated at 15°C, 20°C, 25°C, and 30°C, as shown in Fig 6. The mass ratio of acetic acid to α-pinene was 25:10, which was converted into a molar ratio of 5.67:1. Due to the excess of acetic acid, it could be assumed that the concentration of acetic acid was constant during the reaction. By fitting the experimental data in Fig 6, the relationship between the content of α-pinene and terpinyl acetate and the reaction time can be obtained, as shown in the supporting information (Formulas (S1)–(S8) in S1 File).

The derivative of Formulas (S1)–(S8) in S1 File can be used to obtain the change rate of the content of α-pinene and terpinyl acetate with increasing reaction time, as shown in the supporting information (Formulas (S9)–(S16) in S1 File).

**Fig 7:**
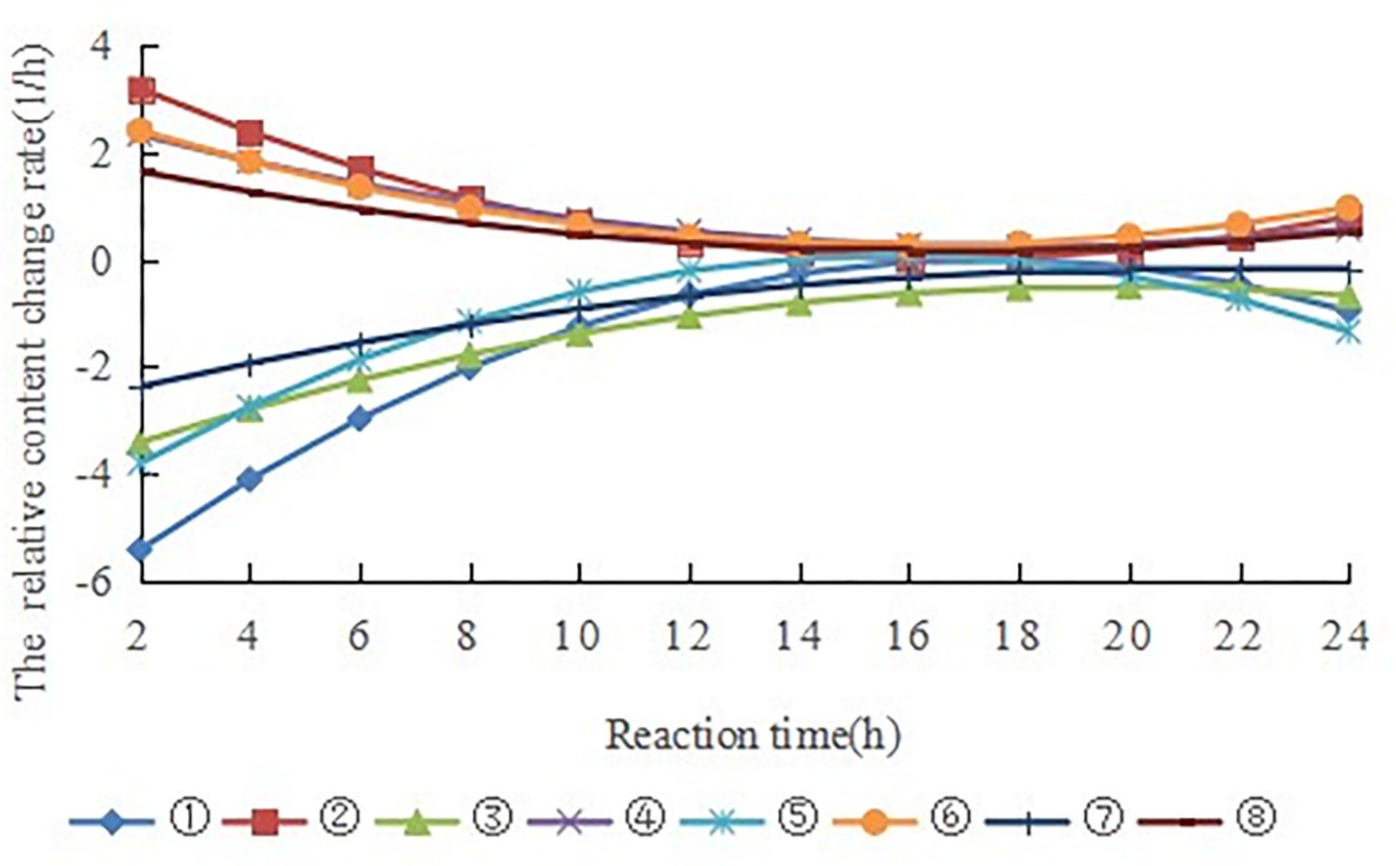
The change rate of the content of α-pinene and terpinyl acetate. Note: ① v1 (15°C); ② v2 (15°C); ③ v1 (20°C); ④ v2 (20°C); ⑤ v1 (25°C); ⑥ v2 (25°C); ⑦ v1 (30°C); ⑧ v2 (30°C). V represents the change rate of the content of α-pinene and terpinyl acetate with increasing reaction time.

According to formulas (S9)–(S16) in S1 File, the change rates of the contents of α-pinene and terpinyl acetate were plotted, as shown in Fig 7. The absolute value of the change rate gradually decreased with increasing reaction time, and the corresponding time at their minimum value was about 18 h. The change rate of the terpinyl acetate content was positive, and it decreased gradually with increasing time. At its minimum value, the formation of terpinyl acetate reached equilibrium. The change rate of α-pinene content was negative, and its absolute value gradually decreased with time. The α-pinene reaction reached equilibrium at its minimum value. In addition to the formation of terpinyl acetate via the reaction of α-pinene and acetic acid, isomerization by-products were also generated. Thus, the time at which α-pinene and terpinyl acetate reached equilibrium was not synchronous, meaning the time at which the change rate of α-pinene content reached the minimum value was greater than that of terpinyl acetate.

### Reaction mechanism of catalyzed terpinyl acetate synthesis from α-pinene

Due to Lewis acidic nature of B2O3 it can react with the HCA hydroxyl group to form a coordinate bond, thereby enhancing the ability of HCA to donate protons. The HCA and boric acid/B2O3 can act as catalysts for terpinyl acetate synthesis from α-pinene, similar to a previous study [3]. Liu et al. [3] found that when an ionic liquid and monochloroacetic acid co-catalyze the reaction between α-pinene and acetic acid, α-pinene was more likely to form unstable oxonium ions with strong monochloroacetic acid after forming carbocations under the catalysis of protonic acid. Then, acetic acid can quickly displace monochloroacetic acid to form terpene acetate.

**Fig 8:**
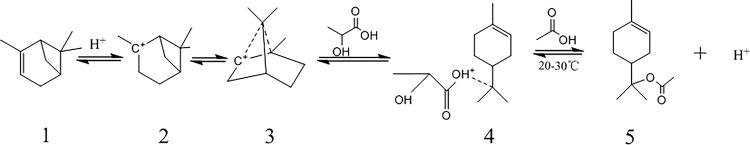
Esterification of α-pinene catalyzed by lactic acid complex catalyst. 1: α-pinene; 2: carbocation; 3: carbocation; 4: oxonium ion; 5: a-terpinyl acetate.

When HCA is catalyzed by boric acid/B2O3 composite, α-pinene forms carbocation (2) under the catalysis of protonic acid. The carbocation can also rearrange to form a different carbocation (3), as well as oxonium ions (4). Then, the acetic acid in the reaction system can rapidly replace HCA to form terpinyl acetate (5), as shown in Fig 8. The carbocation formed after rearrangement then likely reacts with HCA to form an intermediate. Therefore, HCA, with more branches and larger groups, does not favor the formation of oxonium ions. For comparison, citric acid, with more branches, often showed relatively low catalytic activity. In addition, HCA is not easily soluble in α-pinene, resulting in a low reaction. The B2O3 is more likely to form a complex with HCA, with enhanced contact with α-pinene. Therefore, it shows higher catalytic activity. Additionally, acetic acid enhances the contact between HCA and α-pinene to a certain degree; thus, an excess amount of acetic acid is needed for the reaction. Moreover, more isomeric by-products will form if the reaction temperature goes above 30°C.

**Fig 9:**
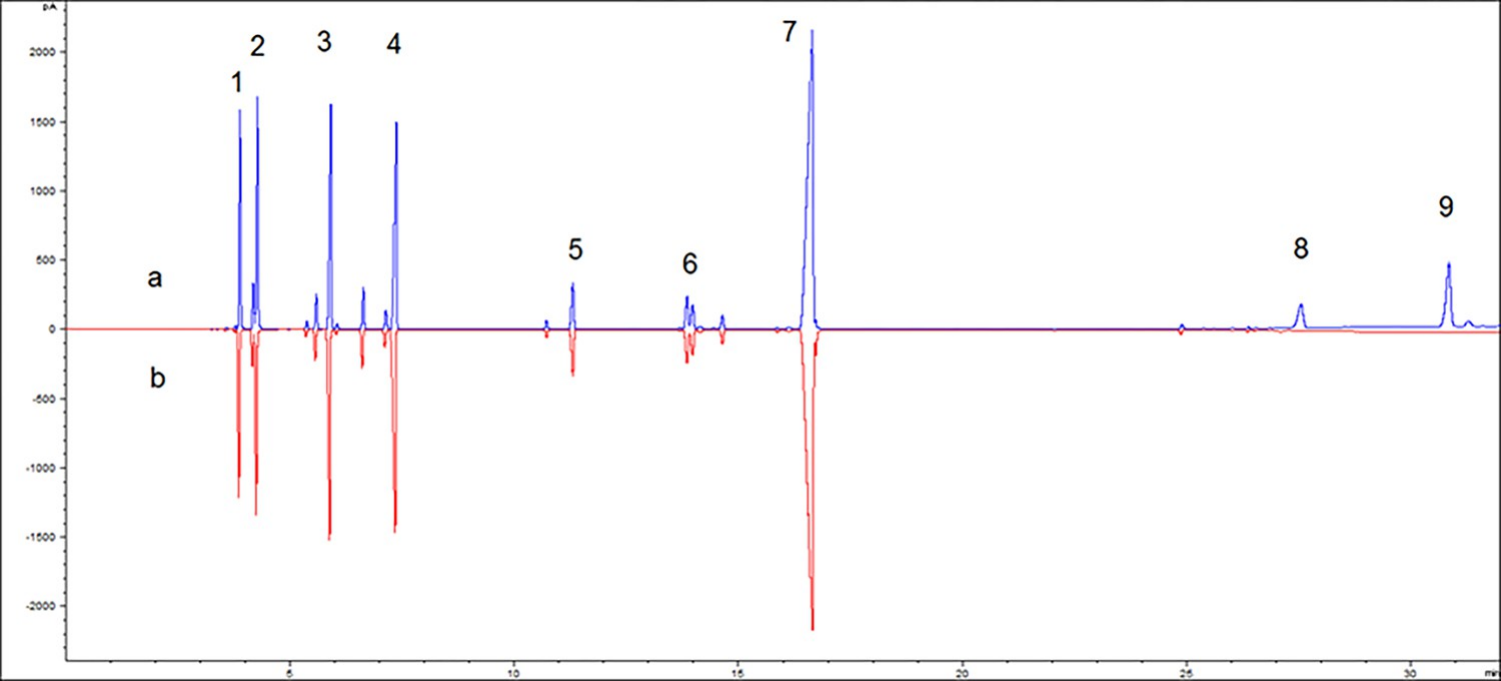
Gas chromatogram of the products generated when lactic acid–B2O3 was used as the catalyst. a) pH-neutral products after being washed with water. b) Products after being washed with 25% sodium hydroxide. Note: 1.α-pinene; 2. limonene; 3. terpinene; 4. isoterpinene; 5. terpineol; 6. bornyl acetate; 7. terpinyl acetate; 8. bornyl lactate; 9. terpinyl lactate.

Lactic acid is a weaker acid, compared to monochloroacetic acid. Considering the dispersing effect of α-hydroxyl on positive charges, lactic acid can help to form more stable oxonium ions. Therefore, when using lactic acid–B2O3 composite catalysts for the synthesis of terpinyl acetate from α-pinene, the by-products contained isobornyl lactate and terpinyl lactate. The isobornyl lactate and terpinyl lactate were removed when washing the product using 25% sodium hydroxide, as presented in Fig 9. The reaction mechanism shown in Fig 8 can be further demonstrated by analyzing the α-pinene esterification product in Fig 9. The gas chromatograms shown in Fig 9A and 9B were the same product. After the reaction, the product was washed to neutral with water as sample a, and then the terpinyl lactate was washed with alkali as sample b. In summary, the HCA composite catalyst showed high catalytic activity. The reaction of boric acid/B2O3 with the hydroxyl group of HCA formed a complex that increased the acidity of HCA, while the α-hydroxyl group of HCA facilitates the formation of stable oxonium ions, thereby diminishing the progress of isomerization side reactions. However, from the above reaction mechanism, we could see that for citric acid containing three adjacent carboxyl groups, the large steric hindrance was not conducive to the formation of onium ions with carbocation 4. Compared with the other three composite catalysts, the citric acid–boric acid composite catalyst had higher acidity but lower catalytic activity for the esterification of α-pinene.

Terpinyl acetate, with a content of 98%, was obtained by vacuum fractional distillation of the reaction product. The ^1^H-NMR spectrum of the synthesized terpinyl acetate is shown in the supporting information (S1 Fig in S1 File). The ^1^H-NMR results were as follows: (400 MHz, CDCl3) δ: 5.37 (t, J = 8.0 Hz, 1H), 2.11–1.91 (m, 6H), 1.90–1.75 (m, 2H), 1.64 (s, 3H), 1.44–1.21 (m, 6H). The mass spectrometry data of terpinyl acetate can be found in the supporting information (S2 Fig in S1 File).

## Experimental section

### Materials and apparatus

The following starting materials and reagents were used in this study: α-pinene (98%), glycolic acid (98%), D-(-)-lactic acid (90%), citric acid (99.5%), L (+)-tartaric acid (99.5%), DL-mandelic acid (99%), boric acid (98%). These compounds were purchased from Macklin and Aladdin (Shanghai, China). Acetic acid (99.5%), citric acid monohydrate (99.5%), ethyl acetate (99.8%) and sodium hydroxide (98%) were purchased from Chengdu Kelong Chemical (Chengdu, Sichuan Province, China).

The reaction apparatus was an organic synthesis unit PPV-3000 (EYELA, Tokyo Rikakikai). In addition, a 7890A gas chromatograph (Agilent, USA), equipped with DB-FFAP quartz capillary chromatography column (60 m × 0.25 mm × 0.25 μm) (Agilent, USA). The TQ456 gas chromatograph was coupled with a mass spectrometer (GC-MS) (Bruker, USA) and a BR-5 elastic quartz capillary column (30 m × 0.25 mm × 0.25 μm). The Avance III 400 MHz nuclear magnetic resonance spectrometer (Bruker, Ferrandon, Switzerland).

### Experimental methods

In the orthogonal experiment, 15 g of α-pinene was used in the reaction. The amounts of acetic acid, HCA, and boric acid and the reaction temperature and time are presented in Table 1. The catalyst was filtered after the reaction. The product was poured into a separatory funnel and neutralized with 5% sodium hydroxide. The upper product was washed three times with water, dried over anhydrous sodium sulfate, and then sampled for analysis. The terpinyl acetate was isolated by vacuum fractionation with a vacuum pressure of −0.085 MPa, and the fraction was collected at 125°C.

In the single factor experiments, 10 g of α-pinene, 25 g of acetic acid, 0.5 to 1 g HCA, 0.3 to 5 g boric acid or B2O3 were added to a reaction flask. The mixture was stirred (500 rpm), and the reaction temperature was maintained at 25 to 28°C. The mixture reacted for 24 h. After the reaction, the catalyst was filtered, and the product was poured into a separatory funnel. Then, 5% sodium hydroxide was added for neutralization. The upper product was washed three times using water, dried using anhydrous sodium sulfate, and then sampled for analysis.

### Analytical methods

The proportions of the starting materials and products were calculated according to the gas chromatography (GC) area normalization method. The conversion of α-pinene and terpinyl acetate selectivity was estimated according to the following formulas, respectively:

$$\mathrm{Conversion}\;\mathrm{of}\;\alpha ‐\mathrm{pinene}=\frac{\mathrm{GC}‐\mathrm{determined}\;\mathrm{content}\;\mathrm{of}\;\alpha ‐\mathrm{pinene}\;\mathrm{before}\;\mathrm{reaction}‐\mathrm{GC}‐\mathrm{determined}\;\mathrm{content}\;\mathrm{of}\;\alpha ‐\mathrm{pinene}\;\mathrm{after}\;\mathrm{reaction}}{\mathrm{GC}‐\mathrm{determined}\;\mathrm{content}\;\mathrm{of}\;\alpha ‐\mathrm{pinene}\;\mathrm{before}\;\mathrm{reaction}}$$ (1)

$$\mathrm{Terpinyl}\;\mathrm{acetate}\;\mathrm{selectivity}=\frac{\mathrm{GC}‐\mathrm{determined}\;\mathrm{content}\;\mathrm{of}\;\mathrm{terpinyl}\;\mathrm{acetate}}{\mathrm{GC}‐\mathrm{determined}\;\mathrm{content}\;\mathrm{of}\;\alpha ‐\mathrm{pinene}\;\mathrm{before}\;\mathrm{reaction}‐\mathrm{GC}‐\mathrm{determined}\;\mathrm{content}\;\mathrm{of}\;\alpha ‐\mathrm{pinene}\;\mathrm{after}\;\mathrm{reaction}}$$ (2)

For GC analysis, high purity nitrogen was used as the carrier gas, and the temperature program was as follows. The initial temperature was 70°C (2 min hold), with the first ramp of 5°C min^‒1^ to 150°C (3 min hold), followed by a second ramp of 10°C min^‒1^ to 230°C (10 min hold). The inlet temperature was set to 250°C, and the total flow rate was 130.5 mL min^‒1^, with a split ratio of 50:1 and a septum purge rate of 3 mL min^‒1^. The analytes were detected using a flame ionization detector (FID), with a detection port temperature of 250°C, a hydrogen flow rate of 40 mL min^‒1^, an air flow rate of 450 mL min^‒1^, and a nitrogen purge rate of 25 mL min^‒1^. The injection volume was 0.2 μL.

The GC-MS conditions were as follows. The carrier gas consisted of high-purity helium gas, and the oven temperature was 50°C (3 min). The temperature increased at 20°C/min to 120°C, 2°C/min to 180°C (2 min), and at 50°C/min to 250°C. It was then held for 5 min. The inlet temperature was 230°C, and the interface temperature was 250°C.

The MS conditions included an EI ion source, an ionization voltage of 70 eV, and a scan range of 45–350 u. The full scan mode employed a solvent delay for 5 min, and the injection volume was 0.5 μL (sample was dissolved in ethanol at a mass fraction of 1%).

For proton nuclear magnetic resonance (1H-NMR) data acquisition, the sample was placed in a sample measuring tube. CDCl3 was added, and the sample was analyzed by a 400-MHz nuclear magnetic resonance (NMR) instrument (frequency: 400 MHz), where the temperature was 23.75°C, the number of scans was 64, and the pulse width was 12.6 μs.

## Conclusions

The HCA–boric acid composite catalyst improved the direct synthesis of terpinyl acetate from α-pinene and acetic acid. Orthogonal experiments indicated the most important factor impacting the content of terpinyl acetate was the HCA amount. The performance of the composite catalyst was related to the pKa1 of HCA. The performance of the composite catalyst followed the trend: tartaric acid–boric acid > glycolic acid–boric acid > mandelic acid–boric acid > citric acid–boric acid > lactic acid–boric acid. When using tartaric acid–boric acid, the α-pinene conversion was as high as 91.8%, and the terpinyl acetate selectivity was 45.6%.

Replacing boric acid with B2O3 improved the activity of the HCA composite catalyst and reduced the amount of HCA required. When the tartaric acid and B2O3 contents were 5% and 3% of the α-pinene mass content, respectively, the content of terpinyl acetate reached 40.8%. When the lactic acid and B2O3 contents were 10% and 4% of the α-pinene mass content, respectively, the content of terpinyl acetate reached 43.0%.

Water reacted unfavorably with the HCA–boric acid composite catalyst, but an appropriate amount of water was beneficial to improve the catalytic activity of HCA–B2O3. When the water content was 1% of the α-pinene mass content, the α-pinene conversion was 89.6%, and the content of terpinyl acetate and selectivity was 41.7% and 47.5%, respectively.

The α-hydroxyl group of HCA helped improve the catalytic activity. However, HCA, with more branches and large groups, was not beneficial to the formation of stable oxonium ions. For example, the catalytic performance of citric acid composite catalysts was poor.

## Supporting information

S1 File (DOCX)
