# Supplementary material for: Synthesis of terpinyl acetate from α-pinene catalyzed by α-hydroxycarboxylic acid–boric acid composite catalyst
Source: PLoS One. 2024 Apr 25;19(4):e0299218. doi: 10.1371/journal.pone.0299218 (PMC11045133; doi:10.1371/journal.pone.0299218)
Supplement: S1 File — (DOCX) [file pone.0299218.s001.docx]

Supporting Information

**S1 Table.** Results of orthogonal experimental on lactic acid–boric acid.

| **No.** | **Temperature (°C)** | **Time (h)** | **Molar ratio of acetic acid to α-pinene** | **Molar ratio of lactic acid to α-pinene** | **Molar ratio of boric acid to α-pinene** | **Conversion of α-pinene (%)** | **Terpinyl acetate content (%)** | **Selectivity (%)** |
| --- | --- | --- | --- | --- | --- | --- | --- | --- |
| 1 | 27 | 20 | 2:1 | 3:50 | 1:500 | 12.4 | 5.0 | 41.2 |
| 2 | 27 | 30 | 3:1 | 1:50 | 7:1000 | 5.7 | 2.6 | 46.4 |
| 3 | 22 | 20 | 3:1 | 1:5 | 1:250 | 39.4 | 15.5 | 40.1 |
| 4 | 33 | 24 | 3:1 | 1:10 | 1:500 | 34.3 | 9.3 | 27.8 |
| 5 | 22 | 15 | 1:1 | 1:50 | 1:500 | 2.7 | 1.4 | 53.0 |
| 6 | 22 | 30 | 2:1 | 1:10 | 1:100 | 28.4 | 11.3 | 40.5 |
| 7 | 33 | 15 | 2:1 | 1:5 | 7:1000 | 65.5 | 22.0 | 34.4 |
| 8 | 22 | 24 | 4:1 | 3:50 | 7:1000 | 16.9 | 7.3 | 44.1 |
| 9 | 40 | 24 | 2:1 | 1:50 | 1:250 | 7.8 | 3.1 | 41.2 |
| 10 | 27 | 24 | 1:1 | 1:5 | 1:100 | 58.0 | 16.5 | 29.0 |
| 11 | 33 | 30 | 1:1 | 3:50 | 1:250 | 26.4 | 7.8 | 30.2 |
| 12 | 27 | 15 | 4:1 | 1:10 | 1:250 | 22.4 | 9.4 | 43.0 |
| 13 | 40 | 15 | 3:1 | 3:50 | 1:100 | 30.8 | 12.2 | 40.4 |
| 14 | 33 | 20 | 4:1 | 1:50 | 1:100 | 8.9 | 4.0 | 46.5 |
| 15 | 40 | 30 | 4:1 | 1:5 | 1:500 | 65.3 | 25.1 | 39.2 |
| 16 | 40 | 20 | 1:1 | 1:10 | 7:1000 | 40.8 | 13.1 | 32.7 |
| K1 | 33.485 | 37.575 | 41.484 | 32.327 | 40.81 |  |  |  |
| K2 | 35.457 | 46.732 | 39.56 | 11.159 | 44.965 |  |  |  |
| K3 | 43.213 | 36.238 | 38.731 | 79.051 | 35.85 |  |  |  |
| K4 | 53.474 | 45.084 | 45.854 | 43.092 | 44.004 |  |  |  |
| k1 | 8.371 | 9.394 | 10.371 | 8.082 | 10.203 |  |  |  |
| k2 | 8.864 | 11.683 | 9.890 | 2.79 | 11.241 |  |  |  |
| k3 | 10.803 | 9.059 | 9.683 | 19.763 | 8.963 |  |  |  |
| k4 | 13.369 | 11.271 | 11.464 | 10.773 | 11.001 |  |  |  |
| Range (R) | 4.997 | 2.624 | 1.781 | 16.973 | 2.279 |  |  |  |
| Rank | 2 | 3 | 5 | 1 | 4 |  |  |  |

**S2 Table.** Results of orthogonal experiments on glycolic acid–boric acid catalyst.

| **No.** | **Temperature (°C)** | **Time (h)** | **Molar ratio of acetic acid to α-pinene** | **Molar ratio of glycolic acid to α-pinene** | **Molar ratio of boric acid to α-pinene** | **Conversion of α-pinene (%)** | **Terpinyl acetate content (%)** | **Selectivity (%)** |
| --- | --- | --- | --- | --- | --- | --- | --- | --- |
| 1 | 27 | 20 | 2:1 | 3:50 | 1:500 | 20.0 | 8.2 | 41.8 |
| 2 | 27 | 30 | 3:1 | 1:50 | 7:1000 | 8.0 | 3.7 | 47.6 |
| 3 | 22 | 20 | 3:1 | 1:5 | 1:250 | 56.5 | 22.9 | 41.5 |
| 4 | 33 | 24 | 3:1 | 1:10 | 1:500 | 39.2 | 16.2 | 42.2 |
| 5 | 22 | 15 | 1:1 | 1:50 | 1:500 | 4.2 | 2.2 | 53.4 |
| 6 | 22 | 30 | 2:1 | 1:10 | 1:100 | 31.9 | 12.9 | 41.2 |
| 7 | 33 | 15 | 2:1 | 1:5 | 7:1000 | 82.3 | 27.3 | 33.9 |
| 8 | 22 | 24 | 4:1 | 3:50 | 7:1000 | 22.2 | 10.3 | 47.1 |
| 9 | 40 | 24 | 2:1 | 1:50 | 1:250 | 11.2 | 4.8 | 43.5 |
| 10 | 27 | 24 | 1:1 | 1:5 | 1:100 | 68.7 | 19.4 | 28.7 |
| 11 | 33 | 30 | 1:1 | 3:50 | 1:250 | 28.1 | 10.8 | 39.2 |
| 12 | 27 | 15 | 4:1 | 1:10 | 1:250 | 34.7 | 15.7 | 46.1 |
| 13 | 40 | 15 | 3:1 | 3:50 | 1:100 | 44.4 | 18.8 | 43.1 |
| 14 | 33 | 20 | 4:1 | 1:50 | 1:100 | 10.3 | 4.9 | 49.1 |
| 15 | 40 | 30 | 4:1 | 1:5 | 1:500 | 81.7 | 31.3 | 39.0 |
| 16 | 40 | 20 | 1:1 | 1:10 | 7:1000 | 49.6 | 16.3 | 33.6 |
| K1 | 46.945 | 52.394 | 53.169 | 47.975 | 57.835 |  |  |  |
| K2 | 48.274 | 58.651 | 61.643 | 15.637 | 57.63 |  |  |  |
| K3 | 59.258 | 50.584 | 48.635 | 100.884 | 54.143 |  |  |  |
| K4 | 71.085 | 63.933 | 62.115 | 61.066 | 55.954 |  |  |  |
| k1 | 11.736 | 13.099 | 13.292 | 11.994 | 14.459 |  |  |  |
| k2 | 12.069 | 14.662 | 15.411 | 3.909 | 14.408 |  |  |  |
| k3 | 14.815 | 12.646 | 12.159 | 25.221 | 13.536 |  |  |  |
| k4 | 17.771 | 15.983 | 15.529 | 15.267 | 13.989 |  |  |  |
| Range (R) | 6.035 | 3.337 | 3.37 | 21.312 | 0.923 |  |  |  |
| Rank | 2 | 4 | 3 | 1 | 5 |  |  |  |

**S3 Table.** Results of orthogonal experiments on mandelic acid–boric acid catalyst.

| **No.** | **Temperature (°C)** | **Time (h)** | **Molar ratio of acetic acid to α-pinene** | **Molar ratio of mandelic acid to α-pinene** | **Molar ratio of boric acid to α-pinene** | **Conversion of α-pinene (%)** | **Terpinyl acetate content (%)** | **Selectivity (%)** |
| --- | --- | --- | --- | --- | --- | --- | --- | --- |
| 1 | 27 | 20 | 2:1 | 3:50 | 1:500 | 24.4 | 9.8 | 41.0 |
| 2 | 27 | 30 | 3:1 | 1:50 | 7:1000 | 9.0 | 4.1 | 46.9 |
| 3 | 22 | 20 | 3:1 | 1:5 | 1:250 | 77.1 | 25.5 | 33.7 |
| 4 | 33 | 24 | 3:1 | 1:10 | 1:500 | 58.4 | 25.8 | 45.1 |
| 5 | 22 | 15 | 1:1 | 1:50 | 1:500 | 4.1 | 1.7 | 41.8 |
| 6 | 22 | 30 | 2:1 | 1:10 | 1:100 | 36.3 | 13.8 | 38.8 |
| 7 | 33 | 15 | 2:1 | 1:5 | 7:1000 | 98.7 | 30.3 | 31.3 |
| 8 | 22 | 24 | 4:1 | 3:50 | 7:1000 | 26.8 | 12.4 | 47.2 |
| 9 | 40 | 24 | 2:1 | 1:50 | 1:250 | 10.3 | 4.7 | 46.5 |
| 10 | 27 | 24 | 1:1 | 1:5 | 1:100 | 75.3 | 22.7 | 30.8 |
| 11 | 33 | 30 | 1:1 | 3:50 | 1:250 | 24.8 | 9.7 | 39.9 |
| 12 | 27 | 15 | 4:1 | 1:10 | 1:250 | 54.3 | 25.3 | 47.6 |
| 13 | 40 | 15 | 3:1 | 3:50 | 1:100 | 37.2 | 17.1 | 46.7 |
| 14 | 33 | 20 | 4:1 | 1:50 | 1:100 | 11.4 | 5.7 | 51.0 |
| 15 | 40 | 30 | 4:1 | 1:5 | 1:500 | 100.0 | 19.8 | 20.2 |
| 16 | 40 | 20 | 1:1 | 1:10 | 7:1000 | 44.6 | 16.0 | 36.7 |
| K1 | 62.007 | 57.056 | 58.589 | 48.973 | 57.050 |  |  |  |
| K2 | 53.333 | 47.406 | 72.472 | 16.222 | 62.824 |  |  |  |
| K3 | 71.431 | 65.561 | 50.126 | 98.227 | 65.227 |  |  |  |
| K4 | 57.576 | 74.324 | 63.16 | 80.925 | 59.246 |  |  |  |
| k1 | 15.502 | 14.264 | 14.647 | 12.243 | 14.263 |  |  |  |
| k2 | 13.333 | 11.852 | 18.118 | 4.0555 | 15.706 |  |  |  |
| k3 | 17.858 | 16.390 | 12.532 | 24.557 | 16.307 |  |  |  |
| k4 | 14.394 | 18.581 | 15.790 | 20.231 | 14.812 |  |  |  |
| Range (R) | 4.525 | 6.730 | 5.587 | 20.501 | 2.044 |  |  |  |
| Rank | 4 | 2 | 3 | 1 | 5 |  |  |  |

**S4 Table.** Results of orthogonal experiments on tartaric acid–boric acid catalyst.

| **No.** | **Temperature (°C)** | **Time (h)** | **Molar ratio of acetic acid to α-pinene** | **Molar ratio of tartaric acid to α-pinene** | **Molar ratio of boric acid to α-pinene** | **Conversion of α-pinene (%)** | **Terpinyl acetate content (%)** | **Selectivity (%)** |
| --- | --- | --- | --- | --- | --- | --- | --- | --- |
| 1 | 27 | 20 | 2:1 | 3:50 | 1:500 | 24.4 | 10.5 | 44.0 |
| 2 | 27 | 30 | 3:1 | 1:50 | 7:1000 | 1.4 | 0.7 | 46.8 |
| 3 | 22 | 20 | 3:1 | 1:5 | 1:250 | 91.8 | 41.0 | 45.6 |
| 4 | 33 | 24 | 3:1 | 1:10 | 1:500 | 96.4 | 31.8 | 33.6 |
| 5 | 22 | 15 | 1:1 | 1:50 | 1:500 | 0.3 | 0.2 | 66.7 |
| 6 | 22 | 30 | 2:1 | 1:10 | 1:100 | 89.3 | 31.7 | 36.2 |
| 7 | 33 | 15 | 2:1 | 1:5 | 7:1000 | 98.9 | 22.4 | 23.1 |
| 8 | 22 | 24 | 4:1 | 3:50 | 7:1000 | 70.8 | 32.5 | 46.9 |
| 9 | 40 | 24 | 2:1 | 1:50 | 1:250 | 29.3 | 12.4 | 43.1 |
| 10 | 27 | 24 | 1:1 | 1:5 | 1:100 | 6.8 | 2.6 | 38.7 |
| 11 | 33 | 30 | 1:1 | 3:50 | 1:250 | 47.9 | 13.7 | 29.2 |
| 12 | 27 | 15 | 4:1 | 1:10 | 1:250 | 94.2 | 37.7 | 40.8 |
| 13 | 40 | 15 | 3:1 | 3:50 | 1:100 | 92.5 | 28.5 | 31.4 |
| 14 | 33 | 20 | 4:1 | 1:50 | 1:100 | 31.3 | 14.8 | 48.3 |
| 15 | 40 | 30 | 4:1 | 1:5 | 1:500 | 99.9 | 10.7 | 10.9 |
| 16 | 40 | 20 | 1:1 | 1:10 | 7:1000 | 91.0 | 19.3 | 21.6 |
| K1 | 51.449 | 85.630 | 77.026 | 85.200 | 53.172 |  |  |  |
| K2 | 105.504 | 56.785 | 101.917 | 28.061 | 74.857 |  |  |  |
| K3 | 82.658 | 79.277 | 35.755 | 76.715 | 104.835 |  |  |  |
| K4 | 70.846 | 88.765 | 95.759 | 120.481 | 77.593 |  |  |  |
| k1 | 12.862 | 21.408 | 19.257 | 21.300 | 13.293 |  |  |  |
| k2 | 26.376 | 14.196 | 25.479 | 7.015 | 18.714 |  |  |  |
| k3 | 20.665 | 19.819 | 8.939 | 19.179 | 26.209 |  |  |  |
| k4 | 17.712 | 22.191 | 23.940 | 30.120 | 19.398 |  |  |  |
| Range (R) | 13.514 | 7.995 | 16.541 | 23.105 | 12.916 |  |  |  |
| Rank | 3 | 5 | 2 | 1 | 4 |  |  |  |

**S5 Table.** Results of orthogonal experiments on citric acid–boric acid catalyst.

| **No.** | **Temperature (°C)** | **Time (h)** | **Molar ratio of acetic acid to α-pinene** | **Molar ratio of citric acid to α-pinene** | **Molar ratio of boric acid to α-pinene** | **Conversion of α-pinene (%)** | **Terpinyl acetate content (%)** | **Selectivity (%)** |
| --- | --- | --- | --- | --- | --- | --- | --- | --- |
| 1 | 27 | 20 | 2:1 | 3:50 | 1:500 | 14.6 | 4.9 | 34.3 |
| 2 | 27 | 30 | 3:1 | 1:50 | 7:1000 | 4.6 | 1.8 | 40.2 |
| 3 | 22 | 20 | 3:1 | 1:5 | 1:250 | 54.8 | 19.7 | 36.6 |
| 4 | 33 | 24 | 3:1 | 1:10 | 1:500 | 28.1 | 10.2 | 37.1 |
| 5 | 22 | 15 | 1:1 | 1:50 | 1:500 | 4.7 | 2.1 | 44.4 |
| 6 | 22 | 30 | 2:1 | 1:10 | 1:100 | 26.7 | 10.1 | 38.7 |
| 7 | 33 | 15 | 2:1 | 1:5 | 7:1000 | 76.2 | 23.5 | 31.4 |
| 8 | 22 | 24 | 4:1 | 3:50 | 7:1000 | 13.3 | 4.8 | 36.7 |
| 9 | 40 | 24 | 2:1 | 1:50 | 1:250 | 6.3 | 2.4 | 37.9 |
| 10 | 27 | 24 | 1:1 | 1:5 | 1:100 | 65.6 | 15.8 | 24.6 |
| 11 | 33 | 30 | 1:1 | 3:50 | 1:250 | 19.3 | 6.4 | 33.8 |
| 12 | 27 | 15 | 4:1 | 1:10 | 1:250 | 24.3 | 8.7 | 36.5 |
| 13 | 40 | 15 | 3:1 | 3:50 | 1:100 | 23.2 | 8.5 | 37.6 |
| 14 | 33 | 20 | 4:1 | 1:50 | 1:100 | 6.3 | 2.4 | 38.5 |
| 15 | 40 | 30 | 4:1 | 1:5 | 1:500 | 80.1 | 27.8 | 35.5 |
| 16 | 40 | 20 | 1:1 | 1:10 | 7:1000 | 45.6 | 14.1 | 31.6 |
| K1 | 31.245 | 41.041 | 40.866 | 24.614 | 45.023 |  |  |  |
| K2 | 36.62 | 46.175 | 40.224 | 8.596 | 44.161 |  |  |  |
| K3 | 42.45 | 33.159 | 38.384 | 86.794 | 37.122 |  |  |  |
| K4 | 52.862 | 42.802 | 43.703 | 43.173 | 36.871 |  |  |  |
| k1 | 7.811 | 10.260 | 10.216 | 6.154 | 11.256 |  |  |  |
| k2 | 9.155 | 11.544 | 10.056 | 2.149 | 11.040 |  |  |  |
| k3 | 10.613 | 8.290 | 9.596 | 21.699 | 9.281 |  |  |  |
| k4 | 13.216 | 10.701 | 10.926 | 10.793 | 9.218 |  |  |  |
| Range (R) | 5.404 | 3.254 | 1.330 | 19.550 | 2.038 |  |  |  |
| Rank | 2 | 3 | 5 | 1 | 4 |  |  |  |

**S6 Table.** pKa1 of different acids.

| **No.** | **HCA** | **Acidity coefficient (25°C), pKa1** |
| --- | --- | --- |
| 1 | Lactic acid | 3.86 |
| 2 | Glycolic acid | 3.83 |
| 3 | Mandelic acid | 3.85 |
| 4 | Tartaric acid | 2.98 |
| 5 | Citric acid | 3.13 |

| $\begin{matrix} \text{y}_{1}\text{(15}℃\text{) =-0.0076x}^{3}\text{+0.3981x}^{2}\text{-6.9255x+65.965} \\ \text{R}^{2}\text{=0.9678 } \end{matrix}$ | (S1) |
| --- | --- |
| $\begin{matrix} \text{y}_{2}\text{(15}℃\text{) =0.0049x}^{3}\text{-0.2457x}^{2}\text{+4.1321x+13.136 } \\ \text{R}^{2}\text{=0.9895} \end{matrix}$ | (S2) |
| $\begin{matrix} \text{y}_{1}\text{(20}℃\text{) =0.0031x}^{3}+\text{0.1834x}^{2}\text{-4.1125x+52.228} \\ \text{R}^{2}\text{=0.9946} \end{matrix}$ | (S3) |
| $\begin{matrix} \text{y}_{2}\text{(20}℃\text{) =0.0028x}^{3}\text{-0.1494x}^{2}\text{+2.9122x+19.291} \\ \text{R}^{2}\text{=0.9836} \end{matrix}$ | (S4) |
| $\begin{matrix} \text{y}_{1}\text{(25}℃\text{) =-0.0069x}^{3}\text{+0.3252x}^{2}\text{-5.0245x+45.001} \\ \text{R}^{2}\text{=0.9438} \end{matrix}$ | (S5) |
| $\begin{matrix} \text{y}_{2}\text{(25}℃\text{) =0.0037x}^{3}\text{-0.177x}^{2}\text{+3.0852x+25.461} \\ \text{R}^{2}\text{=0.9675} \end{matrix}$ | (S6) |
| $\begin{matrix} \text{y}_{1}\text{(30}℃\text{) =0.002x}^{3}+\text{0.1272x}^{2}\text{-2.8599x+34.513 } \\ \text{R}^{2}\text{=0.98566} \end{matrix}$ | (S7) |
| $\begin{matrix} \text{y}_{2}\text{(30}℃\text{) =0.0023x}^{3}\text{-0.1154x}^{2}\text{+2.0293x+27.617} \\ \text{R}^{2}\text{=0.99943} \end{matrix}$ | (S8) |

y_1_ represents the content of α-pinene in the product; y_2_ represents the content of terpinyl acetate; x represents the reaction time; and R is the determination coefficient.

| $\text{v}_{1}\text{(15}℃\text{)=}\frac{\text{dy}_{1}}{\text{dx}}\text{=-0.0229x}^{2}\text{+0.7961x-6.9256 }$ | (S9) |
| --- | --- |
| $\text{v}_{2}\text{(15}℃\text{)=}\frac{\text{dy}_{2}}{\text{dx}}\text{ =0.0146x}^{2}\text{-0.4914x+4.1321}$ | (S10) |
| $\text{v}_{1}\text{(20}℃\text{)=}\frac{\text{dy}_{1}}{\text{dx}}\text{ =-0.0093x}^{2}+\text{0.3668x-4.1125 }$ | (S11) |
| $\text{v}_{2}\text{(20}℃\text{)=}\frac{\text{dy}_{2}}{\text{dx}}\text{ =0.0084x}^{2}\text{-0.2998x+2.9112}$ | (S12) |
| $\text{v}_{1}\text{(25}℃\text{)=}\frac{\text{dy}_{1}}{\text{dx}}\text{ =-0.0206x}^{2}\text{+0.6504x-5.0245 }$ | (S13) |
| $\text{v}_{2}\text{(25}℃\text{)=}\frac{\text{dy}_{2}}{\text{dx}}\text{ =0.011x}^{2}\text{-0.354x+3.0852}$ | (S14) |
| $\text{v}_{1}\text{(30}℃\text{)=}\frac{\text{dy}_{1}}{\text{dx}}\text{ =-0.006x}^{2}+\text{0.2544x-2.8559}$ | (S15) |
| $\text{v}_{2}\text{(30}℃\text{)=}\frac{\text{dy}_{2}}{\text{dx}}\text{ =0.0069x}^{2}\text{-0.2308x+2.0293 }$ | (S16) |

v_1_ represents the change rate of the content of α-pinene in the product with increasing reaction time; v_2_ represents the change rate of the content of terpinyl acetate with increasing reaction time; and x represents the reaction time.


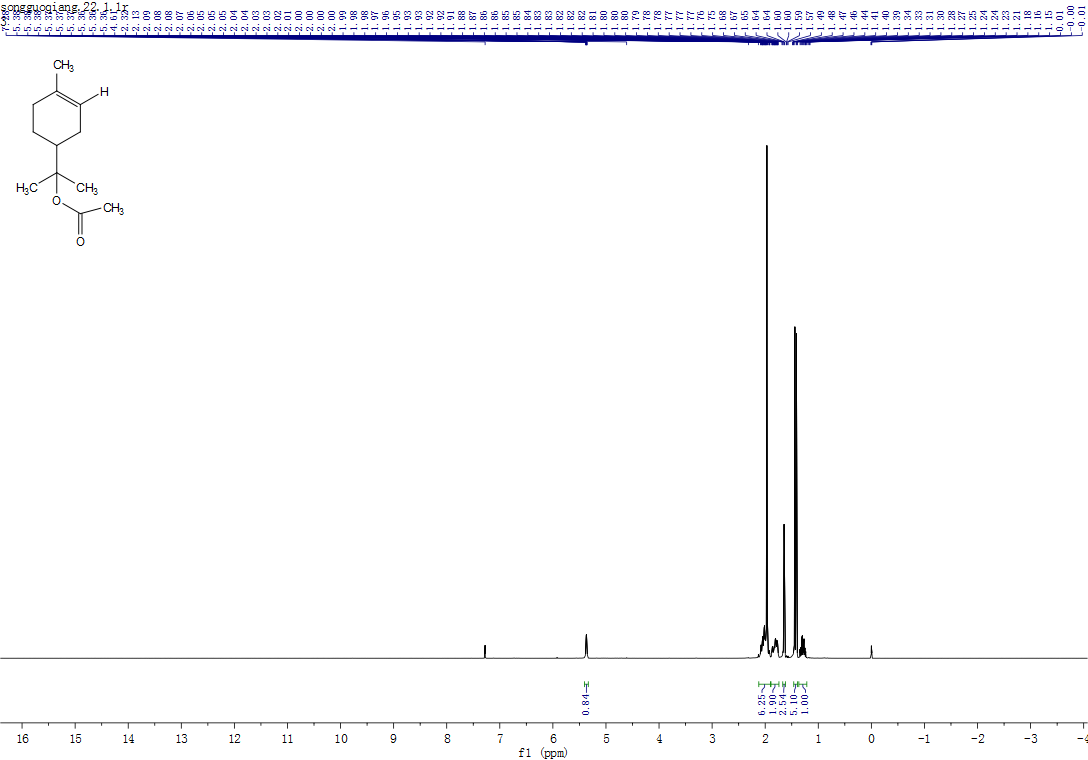


**S1 Fig.** Proton nuclear magnetic resonance (^1^H-NMR) spectra of terpinyl acetate.

**S2 Fig.** The GC-MS of terpinyl acetate.
